# Mediterranean Diet as an Antioxidant: The Impact on Metabolic Health and Overall Wellbeing

**DOI:** 10.3390/nu13061951

**Published:** 2021-06-06

**Authors:** Katherina V. Gantenbein, Christina Kanaka-Gantenbein

**Affiliations:** Division of Endocrinology, Diabetes and Metabolism, First Department of Pediatrics, Aghia Sophia Children’s Hospital, Medical School, National and Kapodistrian University of Athens, 11527 Athens, Greece; katherina.ganten@gmail.com

**Keywords:** Mediterranean diet, antioxidation, metabolic health, reproductive health, gut microbiota, non-communicable diseases, flavonoids, polyphenols, resveratrol, olive oil

## Abstract

It has been established, worldwide, that non-communicable diseases such as obesity, diabetes, metabolic syndrome, and cardiovascular events account for a high percentage of morbidity and mortality in contemporary societies. Several modifiable risk factors, such as sedentary activities, sleep deprivation, smoking, and unhealthy dietary habits have contributed to this increase. Healthy nutrition in terms of adherence to the Mediterranean diet (MD), rich in fruits, legumes, vegetables, olive oil, herbs, spices, and high fiber intake may contribute to the decrease in this pandemic. The beneficial effects of the MD can be mainly attributed to its numerous components rich in anti-inflammatory and antioxidant properties. Moreover, the MD may further contribute to the improvement of reproductive health, modify the risk for neurodegenerative diseases, and protect against depression and psychosocial maladjustment. There is also evidence highlighting the impact of healthy nutrition in female people on the composition of the gut microbiota and future metabolic and overall health of their offspring. It is therefore important to highlight the beneficial effects of the MD on metabolic, reproductive, and mental health, while shaping the overall health of future generations. The beneficial effects of MD can be further enhanced by increased physical activity in the context of a well-balanced healthy lifestyle.

## 1. The Burden of Non-Communicable Diseases

In recent decades, there has been a striking rise in the incidence of non-communicable diseases such as metabolic syndrome, obesity, type 2 diabetes, and non-alcoholic fatty liver disease in modern westernized societies and several countries under development, all predisposing to increased cardiovascular risk. The main factors contributing to the emergence of these diseases are related to people’s diet and behavior, such as the increase in food rich in saturated fat and carbohydrates, including sweetened beverages, lack of physical exercise and smoking. All these factors lead to obesity, hypertension, hyperlipidemia, and insulin resistance [1].

According to the World Health Organization (WHO) non-communicable diseases such as cardiovascular diseases, cancer, respiratory diseases, and diabetes account for 70% of all deaths worldwide. Among the six designated WHO regions, the WHO European region is most severely affected by non-communicable diseases [1].

Several modifiable and non-modifiable factors account for this phenomenon. However, the human genome has not been significantly modified during these last decades and there is growing evidence that epigenetic alterations, mainly due to both intrauterine but also extrauterine insults, may significantly contribute to this increase. Moreover, Western societies have moved from everyday physical activity for walking, jogging, and outdoor leisure to mostly indoor activities, progressively inducing a change in lifestyle with more time spent on sedentary activities. Even the mobility of the modern citizen has shifted from walking, biking, jogging, etc., to sedentary ways of transportation such as driving or using public transport. Furthermore, the widespread use of technology has enabled human communication through all forms of telecommunication, reducing thus the need for face-to-face interaction or meeting outdoors.

This lifestyle change was already established before the outbreak of the COVID-19 pandemic. However, during this pandemic, screen time has experienced a significant increase, since it has become evident that technology has a major contribution in all forms of everyday life activities and has significantly modified many aspects of human behavior. For example, technology has enabled medical consultation through telemedicine, entertainment through all forms of screen time, such as Playstation-based or other technology-based games, f.ex. serious games, or even time spent to watch a ballet or concert or theater action at home. Even the learning process during the lockdown at all levels of education, i.e., primary, in kindergartens and primary schools, secondary, i.e., gymnasium or lyceum or even tertiary level such as university activities has shifted from the classic well-known learning process through teaching in classes, or in amphitheaters in higher education, to a screen-based teaching/educational process. Overall, there has therefore been a significant reduction in the time spent on physical activity and a huge increase in screen time and overall sedentary activities. Therefore, due to this lack of physical activity that the lockdown strategy has imposed during this very recent pandemic, it is of upmost importance to highlight other modifiable factors that may contribute to overall health, such as healthy nutrition.

In addition to the tremendous lack of physical activity during recent years, there is growing evidence that other modifiable risk factors such as smoking, but most importantly, modern nutrition, play a crucial role in the increasing incidence of these non-communicable diseases such as obesity, type 2 diabetes, metabolic syndrome, or non-alcoholic fatty liver disease development, across a wide range of age groups.

Western type diet is characterized by big meals rich in fat and carbohydrate content with a low content in fibers, while sweet beverages significantly contribute to the obesogenic environment, facilitating the development of visceral obesity and insulin resistance. On the contrary, dietary fibers, which have several anti-inflammatory and anti-proliferative actions, are not often represented in the Western diet. When ingested, dietary fibers are not digested in the gastrointestinal tract, but they are fermented by the gut microbiota, leading to the generation of short-chain fatty acids (SCFAs) with anti-inflammatory and anti-aging actions. Furthermore, dietary fibers positively shape the composition of gut microbiota and immunity [2,3].

## 2. The Benefits of the Mediterranean Diet

The important role of a healthy nutrition to combat insulin resistance, obesity, and their deleterious consequences, such as non-alcoholic fatty liver disease (NAFLD), polycystic ovarian syndrome (PCOS), sleep apnea, type 2 Diabetes (T2D) and cardiovascular events, all through the same pathogenetic mechanism of insulin resistance, has become evident through the beneficial effect of the so-called Mediterranean diet (MD). The MD, originally the typical Cretan diet, is mainly a plant-based diet, rich in fruits, vegetables, legumes, and nuts, in association with a moderate consumption of fish and dairy products and a low consumption of red meat and red wine. In addition, herbs, teas, and spices are also highly represented in the MD [4,5,6].

The MD mainly consists of carbohydrates, proteins, and fibers, while it is low in fat content. The most important source of fat in the MD is olive oil, which contains mainly unsaturated fatty acids. As Calabrese et al. mentioned, olive oil also contains 3,3-dimethyl-1-butanol, which prevents the formation of trimethylamine-1-oxide. High levels of trimethylamine-1-oxide are associated with an increased likelihood of cardiovascular events [3]. Furthermore, the consumption of fiber-rich food alters the gut microbiome and enriches the microbiome diversity, which is important for the immune system, possessing anti-inflammatory capacities [7,8]. According to Anderson et al., a diet rich in fibers, such as the MD, is associated with a lower prevalence of cardiovascular diseases, diabetes, metabolic syndrome, and gastrointestinal diseases like gastroesophageal reflux disease, gastric ulcer or diverticulitis [9].

These foods which have beneficial health effects have been called, in the recent literature, functional foods, i.e., foods that have a great contribution in overall health. They contain biologically active nutrients, such as polyphenols which have a significant impact on the prevention and management of chronic non-communicable diseases due to their beneficial anti-oxidative, anti-bacterial or anti-inflammatory effects [10]. Systemic oxidative stress is a hallmark of obesity and metabolic syndrome. Oxidative stress at the cellular level originates from an imbalance between endogenous reactive oxygen species (ROS) production and the natural anti-oxidation system [4]. The molecular structure of polyphenols, important in counteracting oxidative stress, consists of at least two aromatic rings with a hydroxyl group and a carbon bridge between the aromatic rings. They are classified in two groups, the flavonoids, including flavonols, flavones, isoflavones, flavanones, flavan-3-ols, anthocyanidins and dihydrochalcones and the nonflavonoids including phenolic acid, lignans and stilbenes [11].

Moreover, the MD consists of many components rich in mono-unsaturated fatty acid such as oleic acid, in olive oil, omega-3-polyunsaturated fatty acid, such as alpha- linolenic acid in tree nuts, like walnuts, high amounts of flavonoids and antioxidants found in fruits and vegetables and high amounts of dietary fibers that have a great impact on the composition of gut microbiota—all considered to have health-promoting properties and to enhance longevity [12,13]. As already reported above, the significant lack of physical activity that emerged during the lockdown strategy to combat the recent COVID-19 pandemic should be counteracted by a healthier nutrition, such as the MD, Therefore, a review article highlighting the many beneficial effects of MD is especially relevant during this period that is characterized by this shift towards sedentary activities.

## 3. The Impact of Mediterranean Diet on Metabolic Health

### 3.1. The Impact on Obesity Prevention and Management

The increasing prevalence of obesity in westernized societies has become a major public health problem, reaching epidemic dimensions in both adult but also adolescent populations [14,15]. Obesity increase is multifactorial, being the result of lifestyle modifications such as a shift towards sedentary activity and a change from the MD towards a high-fat, carbohydrate-rich Western diet. As highlighted above, oxidative stress is crucially implicated in the chronic inflammation process that is an important component in obesity pathogenesis. Therefore, this chronic inflammation state contributes both to the increased rates of obesity and its complications, such as metabolic syndrome, nonalcoholic fatty liver disease, dyslipidemia, diabetes, all conferring an increased cardiovascular risk [4,14,15]

Therefore, obesity is generally considered as a low-grade inflammation state affecting the whole body [4]. An increased intake of high energy food rich in animal-saturated fat leads to oxidative stress at the cellular level and inflammation by increasing the expression of nicotinamide adenine dinucleuotide phosphate (NADPH) oxidase (NOX) in adipocytes. This triggers adipogenesis and the production of reactive oxygen species. Through the activation of the nuclear factor-kappa B (NF-κB) pathway, it results in an increased expression of the proinflammatory mediators such as tumor necrosis factor alpha (TNFα), interleukin 6 (IL6), leptin while decreasing the secretion of the protective adipokine, adiponectin [4,16]. The increased expression of proinflammatory cytokines leads to downregulation of the anti-inflammatory molecule, AMP-activated protein kinase (AMPK), and the induction of the acute-phase-protein C-reactive protein (CRP) [4].

It has been shown that polyphenols have an anti-inflammatory function. They inhibit proinflammatory molecules and modulate the inflammatory pathways like NF-κB, MAPK and the arachidonic acid pathway [17]. As Li et al. [18] proved, the administration of the flavonoid hesperidin can reduce the secretion of cytokines like interleukin 1 (Il-1) and TNFα and restricts the inflammation in a murine rheumatic arthritis model [18]. Spices are a rich source of polyphenols as well and have anti-inflammatory, anti-carcinogenic and antioxidant capacities. Curcumin, for example, is known for its protective properties against DNA-mutations [19]. Special interest has been laid to resveratrol, a component of grapes, that has been shown to promote intracellular glucose transport and reduce insulin secretion. It has also been shown to inhibit the NADPH-induced oxidative stress and activate the anti-inflammatory molecule peroxisome proliferator-activated receptor gamma (PPARγ) [4]. Moreover, the ingestion of herbal components such as green tea, rich in polyphenols such as epigallocatechin-3-O-gallate (EGCG), contributes to diabetes management and has a lipid-lowering effect, while increasing energy expenditure and decreasing inflammation and oxidative stress through the inhibition of the NF-κB pathway [4]. Furthermore, coffee consumption has been shown to reduce the risk of several diseases such as diabetes mellitus and non-alcoholic fatty liver disease [20]. The mechanism behind this observation could be explained by the polyphenols contained in coffee such as caffeoylquinic acids, trigonelline and N-methylpyridinium. These lead to the upregulation of antioxidant and detoxifying enzymes and the downregulation of proinflammatory mediators via the activation of nuclear factor erythroid 2-related factor 2 (Nrf2) [20]. Obesity is also related with changes in lipid profile [14] with a reduction in high density lipoprotein (HDL) and an increase in triglycerides. Increased visceral adipose tissue is further associated with oxidative stress and it has been shown that telomeres are shorter especially in obesity that is accompanied by metabolic abnormalities leading to metabolic syndrome [21]. Faster shortening of the telomeres and an inhibition of the telomerase activity has therefore been observed in unhealthy obesity [22,23]. In both “healthy” and “unhealthy” obesity, a strategy aiming to reduce oxidative stress at the cellular level can modify morbidity and promote overall health. MD can thus be considered as a natural antioxidant, able to reduce mortality, by reducing the incidence of cardiovascular, metabolic, endocrine, and neurodegenerative diseases through the beneficial effects of its components, mainly polyphenols [24].

Therefore, the effects of MD on the prevention of obesity have simultaneously protective effects on its complications, and it is difficult to discern the specific effects of the different components of the MD on the different expression patterns of inflammation and whether the reduction in insulin resistance may rather contribute to the reduction in metabolic syndrome or merely of type 2 Diabetes, or of nonalcoholic fatty liver disease, since all these non-communicable diseases listed above are interrelated, with many common pathways [4].

### 3.2. The Impact on Diabetes Mellitus

Diabetes mellitus, especially Type 2 Diabetes mellitus (T2DM), is one of the leading diseases in Western societies, in addition to cardiovascular diseases. One of the reasons for the increase in the incidence of diabetes is the high-fat, high-carbohydrate, and low-fiber diet. Consequently, the MD is an important component in the prevention and combat of its pathogenesis. According to Esposito et al., the adherence to a MD has beneficial effects in both the prevention and treatment of diabetes. It has been demonstrated that people following MD had lower hemoglobin A1c (HbA1c) levels compared to the control group. Furthermore, it was observed that fasting glucose could also be reduced by the MD [25]. However, it was not investigated which components of the MD are responsible for this observation. One example could be the fat content. In the MD, more unsaturated fatty acids are consumed, especially as a component of olive oil or table olives, and less saturated fatty acids from animal fat. This has an inhibitory effect on the pathogenesis of diabetes mellitus. Increased monounsaturated fatty acids and reduced saturated fatty acids in the diet have been associated with increased insulin sensitivity and improved beta cell function [26,27]. In addition, the unsaturated fatty acids of olive oil have been shown to increase glucagon-like-peptide-1-(GLP1) expression [28]. GLP1 stimulates insulin secretion and inhibits glucagon secretion. An increased GLP1 expression is also achieved by the SCFAs. As MD is rich in dietary fiber, and the fermentation of this fiber leads to the formation of SCFAs, these then, in turn, stimulate GLP1 expression [26]. Further consideration of the beneficial effects of MD in the pathogenesis of diabetes mellitus could be the widespread use of spices and herbs, also important components of the MD. Zare et al. have been able to show through a randomized control trial (RCT) that cinnamon can improve the glycemic status in patients with diabetes, especially in those with a higher body mass index (BMI) [29]. Cinnamaldehyde, the main component of cinnamon, has also been shown to have lipid-lowering capacities [30]. Furthermore, tea consumption, that is also highly represented in the MD, has health-promoting benefits in the combat against diabetes. Thus, according to Alkhatib et al., the tea has beneficial effects on the pathogenesis of diabetes mellitus by improving glucose metabolism and consequently lowering the fasting glucose level. Moreover, further studies have demonstrated that the consumption of tea is associated with a reduced waist-to-hip ratio and lower levels of blood pressure [10]. The pathomechanism behind the positive effects of these products is still unclear. It could be due to their rich content in polyphenols [4]. There is evidence that glucose uptake is prevented by the inhibition of the enzymes α-amylase and α-glycosidase. Glucose transport by sodium–glucose-linked transporter 1 (SGLT1) could also act as a target for the polyphenols. In addition, there is evidence that polyphenols increase glucose uptake in muscle cells, reduce gluconeogenesis in the liver, and prevent the inflammation-induced destruction of the pancreatic β-cells [31]. An example of these beneficial molecules is the polyphenol quercetin, which reduces the metabolic stress in mitochondria and acts like the widely used antidiabetic drug metformin [32]. Moreover, it can increase glucose uptake through GLUT4 transporter [33]. Another example is rosemary. According to Naimi et al. [34], rosemary contains a large amount of polyphenols, especially carnosic acid, rosmarinic acid and carnosol. These molecules show important antidiabetic effects. They inhibit a-glucosidase and thus intestinal glucose uptake. They also inhibit dipeptidyl peptidase 4, which inactivates the enzyme GLP1. In addition, they inhibit hepatic gluconeogenesis and increase glucose uptake in muscle cells, thus counteracting hyperglycemia. The beneficial properties of rosemary and its polyphenol components have also been confirmed in human studies. As shown in the review by Naimi et al. [34], rosemary also has lipid-lowering and anti-inflammatory effects [34].

### 3.3. The Impact of MD on Non-Alcoholic Fatty Liver Disease

Non-alcoholic fatty liver disease (NAFLD) is one of the leading non-communicable diseases affecting around 20–30% of the general population in Western societies. This increase in incidence occurs in parallel with the increasing incidence of obesity and insulin resistance [35]. According to the “multiple hits” hypothesis, oxidative stress, hypovitaminosis E, low-grade inflammation, and gut microbiota dysbiosis, all contribute to the pathogenesis of NAFLD [36]. A nutraceutical approach in the prevention and management of the disease is the adherence to the MD. MD, rich in nuts with antioxidant properties, may contribute to the decrease in the burden of NAFLD. Although the exact role of the different components of the MD on the prevention and management of NAFLD is not fully elucidated, it became clear that many components have lipid-lowering properties and reduce insulin resistance at the hepatic level, while they have anti-inflammatory and antioxidant properties, further reducing endoplasmic stress. In a randomized control trial (RCT), Yaskolka et al. were able to demonstrate that an MD enriched in green plants with reduced red meat intake was able to enhance intrahepatic fat loss in comparison to the control diet, proving the beneficial effects of MD on reducing hepatic steatosis [37]. Furthermore, in another RCT, Franco et al. were able to demonstrate that a low-glycemic index MD in combination with physical activity was able to reduce the NAFLD score, highlighting the importance of combined lifestyle modifications, including both healthy diet and exercise [38].

### 3.4. The Impact of MD on the Metabolic Syndrome

Metabolic syndrome (MetS) is defined as the simultaneous occurrence of abdominal obesity and at least two of the following: dyslipidemia, mainly characterized by low HDL levels and high triglycerides, and hyperglycemia and/or arterial hypertension. MetS can be further accompanied by hyperuricemia [39], NAFLD, as well as microalbuminuria [40,41]. The presence of the MetS confers a higher risk of cardiovascular events than the addition of these risk factors separately. Furthermore, the MetS is associated with numerous other complications as well as with an increased risk of neoplasia [42,43]. Nowadays, the incidence of MetS is steadily increasing, mainly as a cause of modern lifestyle with increasing obesity rates, and the combination of increased sedentary activities and non-adherence to healthy nutrition. Pathophysiologically, the excess of adipocytes leads to a constant inflammatory state since adipocytes secrete numerous proinflammatory cytokines. This inflammatory state is characterized by the ectopic deposition of lipids, which leads to insulin resistance in muscle cells, liver steatosis with altered metabolism in the liver and beta-cell dysfunction in pancreatic cells and consequently decreased insulin secretory activity that progressively cannot compensate for the increased insulin demands necessary to counteract the peripheral insulin resistance [24].

Kastorini et al. investigated the effects of the MD on metabolism through a meta-analysis. They found that adherence to the MD has a positive effect on metabolism. MD is associated with a lower rate of MetS and its components such as abdominal obesity, low HDL levels and hypertension [44]. The inflammatory state could therefore be a target for the treatment of metabolic syndrome. As already mentioned, the MD has anti-inflammatory effects. An example for these beneficial effects is the tomato, which plays an essential role in the MD. According to Ghavipour et al., the tomato has anti-inflammatory and antioxidant effects [45]. Tomato consumption has been associated with reduced body weight [46]. Olive oil, in turn, as already reported above, can also suppress the inflammatory response. The inflammatory reaction can be further attenuated by the polyphenol quercetin since it induces the secretion of the anti-inflammatory factor, adiponectin, in adipocytes. This leads to a reduction in body weight in obese mice in vivo. Furthermore, it was shown that the administration of quercetin improves the components of the MetS such as dyslipidemia, hypertension, and insulin resistance [47]. In addition, the polyphenol resveratrol was able to inhibit adipocyte proliferation [48,49].

## 4. The Impact on Cardiovascular Disease

Cardiovascular events are mainly due to atherosclerosis and intravascular plaques formation. These plaques can either lead to vascular stenosis and occlusion or break off with the blood flow inducing the formation of a thrombus. Both events subsequently lead to tissue blood underperfusion, tissue ischemia and final necrosis. Cardiovascular disease (CVD) is one of the leading causes of mortality in Western societies. Predisposing factors can be divided into the non-modifiable ones, such as positive family history, age, or male gender, and the modifiable ones, such as smoking, lack of physical exercise and unhealthy diet [50].

An important parameter responsible for the aggravation of atherosclerosis is hyperlipidemia with the combination of high low density lipoprotein (LDL) and low HDL levels. In addition to the high LDL levels, conferring an atherosclerotic cardiovascular risk, it has recently become evident that the susceptibility of LDL particles to aggregate constitutes a further prothrombotic factor and a risk factor for future cardiovascular death. In a very elegant study using data from the Healthy Nordic diet group, Ruuth et al. were able to demonstrate that decreased aggregation susceptibility could be achieved through a diet rich in vitamin E, such as the MD [51]. Furthermore, a study on the effect of a short-term adherence to MD versus fast food (FF) diet was able to achieve a change of HDL lipidome towards a healthier HDL status, further highlighting the cardioprotective effect of MD [52]. Moreover, in vitro and in vivo studies have shown that the consumption of garlic can improve the lipid status, by lowering the level of LDL and increasing the level of HDL. This is achieved by inhibiting enzymes in the pathway of cholesterol synthesis, for example, monooxygenase and 3-hydroxy-3-methyl-glutaryl-coenzyme A reductase (HMG-CoA-reductase). Furthermore, this lowers the blood pressure and inhibits platelet aggregation by inhibiting cyclooxygenase (COX) activity [53]. Thus, the complications of atherosclerosis, such as heart attack or stroke, can be prevented by garlic consumption. An antihypertensive and cardioprotective effect has also been associated with the consumption of olive oil [54]. A diet rich in olive oil consumption, especially of extra-virgin olive oil, allows the intake of phytosterols and polyphenols that have a protective function on the endothelium preventing arteriosclerosis and cardiovascular events. In addition, it increases the expression of anti-inflammatory molecules like PPARγ and reduces the expression of proinflammatory molecules such as IL1β and cyclooxygenase (COX) 2 [4]. The unsaturated fatty acids that are highly represented in the MD and especially in the extra virgin olive oil have been demonstrated, according to the PREDIMED Trial, to contribute to diminished ceramide accumulation and therefore to confer a cardioprotective effect. It is well known that aberrant accumulation of ceramides, that frequently occurs because of excess saturated fat consumption, may lead to impaired cellular function, including impaired insulin function [55], while the MD has a favorable impact on CVD by diminishing ceramide concentration. As mentioned previously, MD contains many biologically active molecules called polyphenols with anti-inflammatory and antioxidant effects [19]. These molecules have also antiplatelet activity [56,57]. Their entire mechanism of action is still unclear [58]. Studies showed that polyphenols, such as oleuropein found in olive oil, reduce oxidative stress, and inhibit the COX1 pathway, which is involved in platelet aggregation [59]. In vitro and in vivo studies showed that polyphenols such as chlorogenic acid or the anthocyanin cyanidin-3-glucoside interrupt thrombin-induced platelet aggregation [60,61]. Another described mechanism of action of polyphenols is the increase in nitric monoxide (NO) levels after consumption of quercetin and catechin [62]. NO has a vasodilating effect and inhibits platelet aggregation [63]. Resveratrol, a polyphenoid that is present in berries, grapes, and grape products such as red wine, has also been proven to have protective effects on the cardiovascular system. It reduces blood pressure, has antiapoptotic effects on cardiomyocytes and prevents thrombus formation [64]. An important complication of hypertension and atherosclerosis is myocardial infarction, in which the occlusion of a blood vessel leads to the reduced perfusion of the myocardium. This leads to remodeling mechanisms that affect both the structure and viability of the myocardium. Nowadays, attempts are being made to prevent these remodeling mechanisms by administering angiotensin converting enzyme (ACE) inhibitors. However, it would be interesting to have a similar effect through natural substances, the so-called neutraceuticals. According to Suzuki et al., catechins, the phenol component of tea, protect against remodeling after myocardial infarction by suppressing proinflammatory cytokine secretion [65]. This anti-inflammatory effect has also been demonstrated in a murine autoimmune myocarditis model, where catechin administration was shown to improve ventricular contractility [66].

## 5. The Impact of MD on Reproductive Health

Healthy nutrition is also important in other areas of overall health, such as reproductive health. It is well known that obesity and its complications are associated with reduced fertility in both women and men [67,68]. Therefore, a healthy low-fat diet such as the MD may have important beneficial effects on reproductive capacity [69]. According to a study by Einarsson et al., overweight women who lost weight before insemination had an increased spontaneous pregnancy rate compared to overweight women who did not lose weight. However, in the group of overweight women who underwent assisted reproduction technologies (ARTs), such as in vitro fertilization (IVF), there was no significant difference in pregnancy rates between women who achieved weight loss and the control group [70]. Therefore, it is important to have a healthy lifestyle from an early age so that obesity can be prevented long before issues such as infertility arise.

### 5.1. The Impact of MD on Female Reproductive Health

Polycystic ovarian syndrome (PCOS) also belongs to the group of metabolic diseases. PCOS is considered as the expression of the metabolic syndrome and insulin resistance at the reproductive axis of the female, although its exact pathomechanism is still unclear. However, obesity and insulin resistance are the main components of its pathogenesis [71]. Women with PCOS have an increased risk of developing type 2 diabetes mellitus. The definition of PCOS includes the fulfillment of the following criteria: hyperandrogenism, either clinical or biochemical, chronic anovulation, expressed as menstrual irregularities, and polycystic appearance of the ovaries on pelvic ultrasound in around 50% of the cases [72]. It is claimed that low-grade inflammation is present in PCOS, which drives the clinical picture [73]. Another parameter that is important in the pathogenesis of PCOS is the presence of advanced glycation end products (AGEs). These substances are either endogenously produced or exogenously supplied through food ingestion [74]. They are mainly the result of the ingestion of high sugar diet and may also be found in animal fat- and protein-rich products and less represented in fruits and vegetables [75]. High AGE levels are associated with increased insulin resistance, that in turn, drives sex-hormone binding globulin (SHBG) reduction and androgen excess. AGEs bind to their receptors (receptor of advanced glycation end products, RAGE), which are also expressed in the ovarian tissue. By binding to RAGE, they induce an inflammatory response that stimulates oxidative stress. AGEs thus accumulate in the ovarian tissue, leading to ovarian dysfunction [74]. Considering all these observations, a dietary modification with a reduced intake of AGEs would be beneficial in PCOS pathology.

Barrea et al. studied how diet differs between women with PCOS and healthy women. They found that women with PCOS follow unhealthier diets with the ingestion of simple carbohydrates, saturated fat, and little fiber content. The constellation of these diets contributes to the inflammatory state and induces oxidative stress [76]. The oxidative stress, in turn, stimulates androgen synthesis in the ovarian tissue. This leads to an increased inflammatory response, so that a vicious circle occurs. In contrast, a healthy high fiber diet with mainly unsaturated and few saturated fatty acids can reduce glucosemia and lipidemia and thus limit the inflammatory response [77]. This oxidative stress can be counteracted through the numerous anti-inflammatory substances contained in healthy diets, as the MD. As Amini et al. have described, polyunsaturated fatty acids have positive effects on insulin sensitivity and can limit hyperandrogenism [78]. Furthermore, Zhang et al. used an Lipopolysaccharide (LPS)-induced sepsis model to investigate the effect of the ω3 polyunsaturated fatty acid-derived metabolite resolvin. It was observed that resolvin leads to a downregulation of proinflammatory mediators and thus to a limitation of the inflammatory response [79]. The lipid-lowering and anti-inflammatory effects of ω3 fatty acids can hence be used as a treatment method in PCOS patients. These molecules are found in fish and olive oil, nutrients abundantly consumed in MD [78]. In addition to vegetables, fish and olive oil, herbs and spices are also frequently used in MD. According to Ashkar et al., these products also have beneficial effects in limiting PCOS pathology. Studies have shown that cinnamon, for example, reduces insulin resistance and fasting insulin levels. Green tea stimulates metabolism and has a lipolytic effect. Chamomile has positive effects on weight control and lowers insulin levels. In murine PCOS models, green tea and chamomile were shown to reduce the number of ovarian cysts observed in pelvic ultrasound. Mint and licorice counteract hirsutism, a common sign of PCOS due to hyperandrogenism. Fennel lowers testosterone levels. Finally, Marrubium vulgare lowers cholesterol, glucose, and oxidative stress [80].

Furthermore, in PCOS, insulin has a stimulatory and anti-apoptotic effect on the theca cells, resulting in the hyperplasia of these cells [81]. According to Wong, the polyphenol resveratrol has proapoptotic properties on the theca cells and can thus antagonize the insulin effect [82]. In addition, resveratrol has been shown to decrease the level of proinflammatory cytokines, such as Interleukin 6, Interleukin 18, TNFα and the acute-phase-protein CRP, which are all markers of the inflammatory state in PCOS. Furthermore, Brenjian et al. described that resveratrol can modulate the stress of the endoplasmic reticulum, also involved in the pathogenesis of PCOS [83]. In addition, this leads to an induction of the expression of SIRT1, which also seems to have protective effects against oxidative stress in the ovarian tissue [84]. It has also been shown that resveratrol decelerates ovarian ageing in rodent experiments and thus increases fertility [85]. A further polyphenol with beneficial properties in the pathogenesis of PCOS seems to be quercetin. It is known that PCOS patients have lower levels of adiponectin and adiponectin receptors. As widely known, adiponectin is beneficial for metabolism as it improves glucose homeostasis and has lipid-lowering effects. Rezvan et al. investigated how the expression levels of adiponectin and its receptors change after quercetin supplementation in PCOS patients. They found that quercetin induces an upregulation of these proteins and could therefore be used as a treatment for PCOS [86,87].

### 5.2. The Impact on Male Reproductive Health

Male fertility is also influenced by lifestyle. Oxidative stress, for example, can contribute to male infertility. A high amount of reactive oxygen species is associated with reduced sperm quality, vitality, and concentration as well as DNA damage in spermatocytes. Oxidative stress is induced by both internal and external factors. Internal factors can be the result of cryptorchidism or testicular inflammation. External factors that lead to enhanced oxidative stress include obesity, smoking and an unhealthy diet [88]. According to Salas-Huetos et al., oxidative stress can be reduced by the antioxidant properties of fruits and vegetables, since these antioxidants act as scavengers and reduce reactive oxygen species [89,90]. Another parameter associated with impaired male fertility is the presence of xenoestrogens, which are found nowadays in increased amounts in meat products [90]. Xenoestrogents are shown to reduce the ejaculate volume, the sperm motility and vitality [91]. Furthermore, it has been observed that increased consumption of trans lipids is associated with reduced sperm quality and lower testosterone levels. In contrast, the polyunsaturated fatty acids found in fish and olive oil or nuts are associated with better sperm quality and morphology [92]. The effects of dyslipidemia on fertility were studied in several animal experimental models. Saez Lancellotti et al. studied how a diet high in cholesterol alters sperm quality. The results showed that the high cholesterol diet led to the formation of abnormal sperm and reduced sperm motility [93]. These observations were significantly reversed after olive oil supplementation [94]. Altogether, these observations support the notion that adherence to the MD would be beneficial in male reproductive issues. The mechanism behind this is most likely due to the polyphenols. According to Simas et al. the polyphenol resveratrol improves male fertility, by ameliorating sperm mobility and functionality. It has been further demonstrated that the diabetes complications on the reproductive system such as DNA fragmentation and disturbed sperm acrosome integrity can be reduced by the ingestion of resveratrol [95]. Resveratrol can also improve male fertility rates in the procedure of cryoconservation since it protects the sperm from oxidative stress [96]. The polyphenols, mainly catechins, contained in green tea also have a protective effect on sperm quality. They protect against spontaneous mutations and chromosomal aberrations. According to Rahman et al., polyphenols may therefore improve fertility through their anti-inflammatory and antioxidant effects [88].

## 6. Transgenerational Effects on the Offspring

Healthy diet already before pregnancy, but mainly during pregnancy and lactation, has a major impact on the metabolic and overall health of the offspring [97,98,99]. As described in the review by Amati et al., maternal nutrition before and during pregnancy has a major impact on fetal health greatly contributing to the gut microbiota diversity of the offspring [97,100]. It was observed that children whose mothers followed MD had a lower risk of developing congenital heart defects. Moreover, intrauterine development is also positively influenced by the MD [97]. Following an MD during pregnancy is associated with a lower rate of small for gestational age (SGA) neonates [98]. In addition, newborns whose mothers followed MD during pregnancy showed a lower probability of developing neural tube defects. Furthermore, it has been demonstrated that maternal diet during pregnancy also affects the rate of congenital malformations, like gastroschisis [99]. Adherence to the MD is further associated with a reduced likelihood of gestational diabetes and hypertensive pregnancy diseases [100]. In contrast, the risk of developing gestational diabetes is increased in overweight women, which also entails many complications for the newborn, including both adverse metabolic health [100] and future neurodevelopment [99,101]. Furthermore, the high body weight of the mother is associated with lower fertilization rate and miscarriage [99]. Moreover, the healthy diet of a pregnant woman rich in dietary fiber shapes the gut microbiota of her offspring [100]. The gut microbiota composition is further affected by the diet of the newborn, since babies fed by infant formulas have a higher number of pathogenic bacteria in comparison to breast-fed babies [102]. However, the positive effects of MD are not limited to the pregnancy and early postnatal period. It has been shown that children whose mothers had a high adherence to the MD are less likely to develop atopic diseases and behavioral problems [97].

## 7. Impact on Autoimmune Disease

Autoimmune diseases are another type of diseases for which a positive impact can be achieved through dietary changes. As an example, we focus on Type 1 diabetes mellitus (T1DM), an autoimmune disease where the autoimmune destruction of the pancreatic beta cells take place, so that insulin synthesis and secretion progressively diminish, up to complete exhaustion. The disease is mainly manifested in early childhood but can also be diagnosed in later ages, and if not appropriately managed through the years, is associated with severe complications, especially micro- and macrovascular events. The pathogenesis of T1DM has not been fully elucidated yet. It is considered that a complex pathogenesis is responsible for its occurrence, notably the combination of inherent genetic predisposing factors with external environmental triggers such as viral infections or dietary components contributing to the autoimmune pathogenesis of the insulitis [103]. Studies have shown that patients with T1DM have an altered intestinal flora compared to healthy individuals [104]. It can begin in infancy or even earlier in life, where it has been shown that breastfed children of mothers who eat a low meat diet have a lower risk of developing T1DM [105]. A high fat diet leads to the colonization of the intestine of mainly Bacteroides species. In contrast, a high fiber diet induces a microbiome rich in Prevotella species [106]. A gut microbiome rich in Bacteroides is correlated with a T1DM-associated intestinal dysbiosis. The type of dominant bacterial species has an influence on the synthesized SCFAs. Beneficial SCFAs, such as butyrates, have immunomodulatory effects and suppress inflammatory responses. In contrast, Bacteroides-dominant gut flora results in the formation of proprionate, succinate and acetate, which have proinflammatory effects and promote autoimmunity by increasing the epithelial permeability of the gut [102]. As already described, this intestinal flora is strongly influenced by nutrition. According to Yang et al., the consumption of a polyphenol-rich diet, such as the MD, increases the amount of beneficial bacterial species and suppresses the growth of pathogenic bacterial species [107].

There is also evidence for the positive effects of the MD in other autoimmune diseases. One example is multiple sclerosis, an autoimmunological disease characterized by immune cell recruitment, mainly T cells, in the central nervous system, and subsequent demyelination. It has been observed that the consumption of saturated fatty acids and especially long-chain fatty acids is associated with an aggravation of the symptomatology and an increase in immune cell recruitment [108]. In contrast, SCFAs have been shown to have an immunomodulatory effect [109]. It has been further suggested that flavonoids have a remyelination-promoting effect [110,111].

## 8. Impact on Neurodegeneration and Mental Health

### 8.1. Polyphenols against Neurodegeneration

Unhealthy diets also have effects on neurodegeneration. The high-fat, low-fiber Western diet has been associated with an increased risk of Parkinson’s disease. The lack of dietary fiber leads to an excess of lipopolysaccharide-producing bacteria in the microbiome, resulting in damage to the intestinal barrier and mitochondrial function [112]. Furthermore, fats are thought to increase the permeability of the blood–brain barrier and increase the quantity of β-amyloid plaques deposition. Polyphenols instead lead to an improvement in cognition, which can be shown by both cognitive tests and functional MRI (fMRI) studies. According to Rajaram et al., the consumption of fruits, green tea, and nuts, which are all important components of the Mediterranean diet, has neuroprotective effects [113]. These effects could be explained by the antioxidant effect of polyphenols [114]. Hesperidin, for example, which is found in citrus fruits, scavenges free oxygen radicals. Furthermore, it suppresses the formation of proinflammatory cytokines and thus also has an anti-inflammatory effect. In addition, according to several studies, it has been shown that hesperidin increases cerebral blood flow, which further prevents cognitive decline. The neuroprotective effect of hesperidin was observed in both animal experiments and in human studies [115]. Hesperidin is just one example of the multitude of polyphenols contained in the MD with neuroprotective properties. Other bioactive compounds are anthocyanins and carotenoids, which are abundantly found in fruits and vegetables. These molecules reduce oxidative stress, by lowering the number of free radicals. Further studies have shown that anthocyanins and the compound of olive oil, olecanthal, prevent the aggregation of Aβ amyloids and tau protein [116]. Isocyanates found in cruciferous vegetables and olecanthal act as natural COX inhibitors and thus limit inflammatory responses [117,118,119]. These findings are based both on in vitro and in vivo studies. The research group of Yammine et al. investigated the effect of phenols on murine N2 neuronal cells after treatment with 7-ketocholesterol. 7-ketocholesterol is an oxidation product that is found in high concentration in neurodegenerative diseases. It promotes oxidative stress, apoptosis, autophagy, mitochondrial and peroxisomal dysfunction. These effects were reduced after the treatment of N2 cells with the polyphenols, resveratrol, quercetin and apigenin [120]. In summary, it can be said that polyphenols, and thus also the MD, have a preventive effect against neurodegenerative diseases such as Alzheimer’s or Parkinson’s disease.

### 8.2. The Impact on Mental Health

The current COVID-19 pandemic has exacerbated neuropsychological disorders, since the lockdown strategy led to significant lifestyle changes that were accompanied by increased social isolation, uncertainty for the future and unemployment. The main consequence of all these changes is depression [121]. Depression is nowadays one of the leading causes of disability [122]. Therefore, it is extremely important to find methods to relieve the symptoms, especially in a natural way, since antidepressant medication has often multiple side effects. One example could be healthy food intake. As already described, a healthy diet helps to combat the pathogenesis of many diseases. The same holds true for depression. As Riera-Sampol et al. have shown, both adherence to MD and a low BMI are beneficial against depression. These positive properties of MD on depression are probably due to nutrients such as unsaturated fatty acids and polyphenols [123]. In an RCT, Parletta et al. investigated how symptoms of depression change based on one’s diet. They showed that the group that followed MD had less stress and negative emotions and scored better on depression scores. To determine the beneficial component of MD, they also studied how the amount of omega-3 and omega-6 polyunsaturated fatty acids was related to the clinical picture. They found that high omega-3 and low omega-6 polyunsaturated fatty acids were associated with a more favorable outcome [124]. The importance of n3-polyunsaturated fatty acids in the pathology of depression was also confirmed by further studies. In addition, comparing n3-polyunsaturated fatty acids to placebo as a treatment of depression also demonstrated the beneficial effects of n3-polyunsaturated fatty acids [125]. These results can probably be attributed to the anti-inflammatory and immunomodulatory properties of n3-polyunsaturated fatty acids since depression is associated with an inflammatory state [126]. Further substances that are associated with a reduced risk of developing depression are polyphenols [127]. The uptake of polyphenols mainly occurs through the intestinal flora, a process that creates an interaction between polyphenols and intestinal flora. Polyphenols regulate the composition of gut bacteria by stimulating the growth of beneficial bacteria and inhibiting the growth of harmful bacteria. The bacteria break down the polyphenols and produce polyphenol metabolites and polyphenol-related bacteria metabolites. SCFAs are an example of bacterial metabolites. The formation of SCFAs induces the secretion of hormones, including leptin, which showed beneficial effects on depressive symptoms in animal experiments [11]. Furthermore, polyphenols can have a positive impact on the homeostasis of neurotransmitters. In patients suffering from depression, a reduced level of dopamine and serotonin has been observed. According to Gu et al., the polyphenol resveratrol modulates this, by increasing the levels of both dopamine and serotonin in a dose-dependent manner. In addition, it increases the level of the neuroprotective molecules brain-derived nerve growth factor (BDNF) and neuropeptide Y (NPY), which are also found to be decreased in patients with depression [128].

## 9. Possible Adverse Effects of Mediterranean Diet

**Figure 1:**
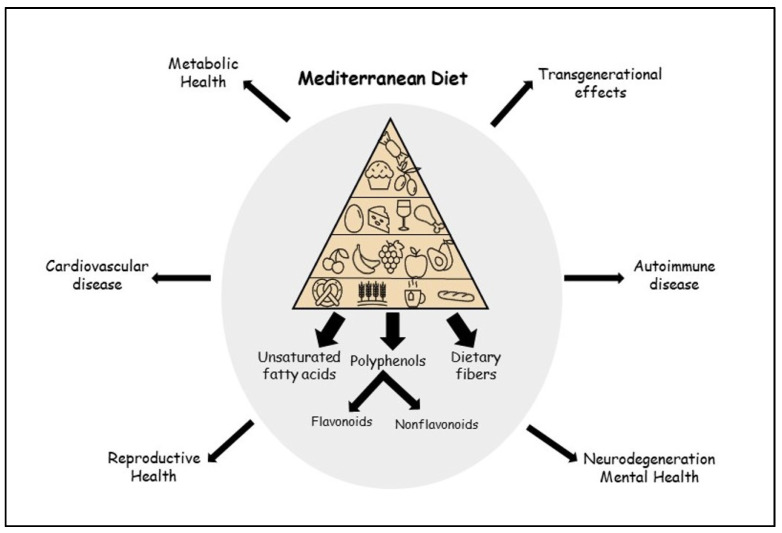
Beneficial effects of the Mediterranean diet.

In addition to the beneficial effects of MD and the polyphenols it contains, adverse effects have also been described. One example is the polyphenol resveratrol. According to Shaito et al., there are indications that the anti-inflammatory effect is dose dependent. A high-dose administration of resveratrol in animal experiments showed proinflammatory and cytotoxic effects. In addition, pharmacokinetic interactions have been described, mainly due to CYP450 induction, which may affect the pharmacokinetics of other drugs [129]. EGCG present in tea has also been associated with adverse effects when administered at high concentrations. A hepatotoxic effect with an increase in hepatic transaminase has been reported in animal experiments as well as in humans. This led to a market withdrawal of high dose-EGCG-supplements [130]. In conclusion, the adverse health effects of polyphenols have mainly been described in the context of an excess supraphysiological intake that does not correspond to the natural diet. Thus, in a healthy balanced diet, the numerous beneficial effects outweigh by far the putative negative effects. In Figure 1, the numerous beneficial effects of MD and its components are illustrated, affecting most aspects of human life and health.

## 10. Conclusions

It has been established, worldwide, that non-communicable diseases, such as obesity, diabetes, non-alcoholic fatty liver disease, metabolic syndrome, and cardiovascular events account for a high percentage of morbidity and mortality in modern westernized societies. Several modifiable factors, such as increase in sedentary activities, sleep deprivation, smoking, and unhealthy dietary habits have significantly contributed to this increase. Healthy nutrition in terms of adherence to the Mediterranean diet, rich in fruits, legumes, vegetables, olive oil, nuts, herbs, spices, and high fiber intake with a reduction in processed red meat intake may contribute to the decrease in this pandemic. Moreover, it seems that MD may further contribute to reproductive health in both men and women, since higher rates of subfertility have been noted nowadays in westernized societies and may further contribute to the overall health of future generations. Moreover, healthy nutrition may modify the risk for neurodegenerative diseases such as Alzheimer disease, or Parkinson’s disease, or even protect against depression and psychosocial maladjustment. There is a lot of evidence highlighting the impact of healthy nutrition of the woman on the composition of gut microbiota and metabolic health of her offspring and may further protect from congenital malformations or even adverse neurodevelopment in the offspring. It is important to highlight all these beneficial effects of the MD and specifically the protective components of the MD such as polyphenols, poly-unsaturated fatty acids, flavonoids, terpenoids, all with anti-inflammatory and antioxidant properties, enhancing metabolic, reproductive, and mental health, and shaping the overall health of future generations. The beneficial effects of a healthy nutrition can be further enhanced by increased physical activity and the avoidance of sleep deprivation and excess psychosocial stress in the context of a healthy lifestyle modification.

## Data Availability

This is a review article, not presenting any unpublished data. All articles used are properly cited in the references.
